# Supplementary material for: Empirical methods for controlling false positives and estimating confidence in ChIP-Seq peaks
Source: BMC Bioinformatics. 2008 Dec 5;9:523. doi: 10.1186/1471-2105-9-523 (PMC2628906; doi:10.1186/1471-2105-9-523)
Supplement: Additional File 1 — A variety of html documents from the USeq web site detailing the available applications, their best usage, output file type descriptions, command line menus, etc. [file 1471-2105-9-523-S1.zip › USeqUserGuides/JavaDoc.zip!/JavaDoc/edu/utah/seq/data/package-tree.html]

edu.utah.seq.data Class Hierarchy


|  |  |  |
| --- | --- | --- |
| **Overview** \| **Package** \| Class \| Use \| **Tree** \| **Deprecated** \| **Index** \| **Help** | |  |
| **PREV**   **NEXT** | **FRAMES**    **NO FRAMES**     **All Classes** |  |


---


## Hierarchy For Package edu.utah.seq.data

**Package Hierarchies:**: All Packages

---

## Class Hierarchy

- java.lang.Object
  - edu.utah.seq.data.**ComparatorEnrichedRegionPosition** (implements java.util.Comparator<T>)- edu.utah.seq.data.**ComparatorEnrichedRegionScore** (implements java.util.Comparator<T>)- edu.utah.seq.data.**ComparatorInfoPoint** (implements java.util.Comparator<T>)- edu.utah.seq.data.**ComparatorPointPosition** (implements java.util.Comparator<T>)- edu.utah.seq.data.**ComparatorSmoothingWindowPosition** (implements java.util.Comparator<T>)- edu.utah.seq.data.**ComparatorSmoothingWindowScore** (implements java.util.Comparator<T>)- edu.utah.seq.data.**ComparatorSmoothingWindowTwoScore** (implements java.util.Comparator<T>)- edu.utah.seq.data.**DividePointData**- edu.utah.seq.data.**EnrichedRegion** (implements java.io.Serializable)- edu.utah.seq.data.**FilterPointData**- edu.utah.seq.data.**HeatMapMaker**- edu.utah.seq.data.**HeatMapMakerPosNeg**- edu.utah.seq.data.**Info** (implements java.io.Serializable)- edu.utah.seq.data.**Point**
                              - edu.utah.seq.data.**InfoPoint**
                              - edu.utah.seq.data.**PointData**- edu.utah.seq.data.**Simulator**- edu.utah.seq.data.**SmoothingWindow** (implements java.io.Serializable)- edu.utah.seq.data.**SmoothingWindowInfo** (implements java.io.Serializable)- edu.utah.seq.data.**SubSamplePointData**

---


|  |  |  |
| --- | --- | --- |
| **Overview** \| **Package** \| Class \| Use \| **Tree** \| **Deprecated** \| **Index** \| **Help** | |  |
| **PREV**   **NEXT** | **FRAMES**    **NO FRAMES**     **All Classes** |  |


---
